# The role of glucocorticoids in the induction of zinc-*α*_2_-glycoprotein expression in adipose tissue in cancer cachexia

**DOI:** 10.1038/sj.bjc.6602404

**Published:** 2005-02-15

**Authors:** S T Russell, M J Tisdale

**Affiliations:** 1 Pharmaceutical Sciences Research Institute, Aston University, Birmingham B4 7ET, UK

**Keywords:** cancer cachexia, glucocorticoids, lipolysis, zinc-*α*_2_-glycoprotein

## Abstract

Loss of adipose tissue in cancer cachexia in mice bearing the MAC16 tumour arises from an increased lipid mobilisation through increased expression of zinc-*α*_2_-glycoprotein (ZAG) in white (WAT) and brown (BAT) adipose tissue. Glucocorticoids have been suggested to increase ZAG expression, and this study examines their role in cachexia and the mechanisms involved. In mice bearing the MAC16 tumour, serum cortisol concentrations increased in parallel with weight loss, and the glucocorticoid receptor antagonist RU38486 (25 mg kg^−1^) attenuated both the loss of body weight and ZAG expression in WAT. Dexamethasone (66 *μ*g kg^−1^) administration to normal mice produced a six-fold increase in ZAG expression in both WAT and BAT, which was also attenuated by RU38486. *In vitro* studies using 3T3-L1 adipocytes showed dexamethasone (1.68 *μ*M) to stimulate lipolysis and increase ZAG expression, and both were attenuated by RU38486 (10 *μ*M), anti-ZAG antibody (1 *μ*gml^−1^), and the *β*3-adrenoreceptor (*β*3-AR) antagonist SR59230A (10 *μ*M). Zinc-*α*_2_-glycoprotein also increased its own expression and this was attenuated by SR59230A, suggesting that it was mediated through the *β*3-AR. This suggests that glucocorticoids stimulate lipolysis through an increase in ZAG expression, and that they are responsible for the increase in ZAG expression seen in adipose tissue of cachectic mice.

Cancer cachexia is characterised by progressive loss of adipose tissue, which can result in the depletion of 85% of the adipose tissue mass, when the weight loss reaches 30% (Fearon, 1992). This loss of fat seems to arise from an increased lipolysis, since both glycerol and fatty acid turnover has been shown to be elevated by 25%, and is similar to the rate observed in patients with severe burns (Legaspi *et al*, 1987). A potential mediator of this increased lipolysis is a lipid-mobilising factor (LMF), which has been isolated both from a cachexia-inducing murine adenocarcinoma (MAC16), and from the urine of patients with cancer cachexia, and has been shown to be identical with the plasma protein zinc-*α*_2_-glycoprotein (ZAG) (Todorov *et al*, 1998). Both LMF and ZAG induce lipolysis directly in isolated adipocytes by a cyclic AMP-mediated process (Hirai *et al*, 1998) and this is initiated through binding to a *β*3-adrenoreceptor (AR) (Russell *et al*, 2002). Although it was originally thought that LMF/ZAG was produced by certain tumours and circulated to adipose tissue, recent results (Bing *et al*, 2004) have shown that ZAG is a natural adipocyte factor found in both white (WAT) and brown (BAT) adipose tissue. During weight loss in mice bearing the MAC16 tumour, both ZAG mRNA and protein increased with increasing weight loss, with a 10-fold increase in ZAG mRNA and protein in WAT, and a 20-fold increase in ZAG protein expression in BAT, at a weight loss of 24%. Dexamethasone markedly increased levels of ZAG mRNA in 3T3L1 adipocytes, as has previously been reported in human breast cancer cells (Lopez-Boado *et al*, 1994). Expression was also increased by the selective *β*3-AR agonist BRL 37344, suggesting that the sympathetic system may also play a role in the regulation of ZAG expression.

Fasting levels of serum cortisol have been shown to be significantly higher in weight-losing patients with stage IV breast cancer than those who have not lost weight (Knapp *et al*, 1991). This suggests that changes in serum cortisol may be responsible for the changes in ZAG expression in WAT and BAT during the development of cachexia. The 5′-flanking region of the human ZAG gene contains a TATA box, a CAT box, an octamer sequence, and three possible Sp1-binding sites, which may be involved in transcriptional regulation of this protein (Ueyama *et al*, 1993). Glucocorticoids have been shown to increase ubiquitin expression in muscle cells through Sp1-binding sites in DNA (Marinovic *et al*, 2002), and this may indicate a mechanism for the increased ZAG gene expression.

This study investigates the role of glucocorticoids in ZAG expression in WAT and BAT during the development of cachexia and investigates the mechanisms involved.

## MATERIALS AND METHODS

### Animals

**Figure 1:**
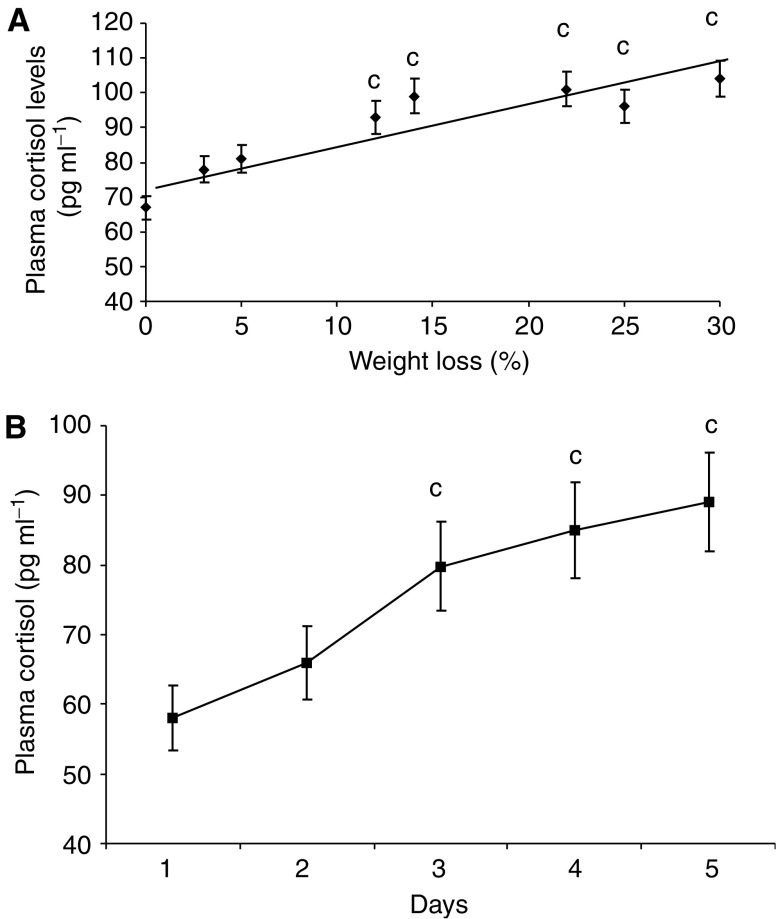
(**A**) Plasma cortisol concentrations in mice bearing the MAC16 tumour with varying extents of weight loss. Blood was removed from animals by cardiac puncture under terminal anaesthia as described in Materials and Methods. Number of animals used for each point, *n*=3. The data fit to a straight line relationship (*r*^2^=0.95). (**B**) Plasma cortisol concentrations in tail bleed samples from MAC16 tumour-bearing mice with time after day 11 after transplantation when weight loss occurred (day 1 in the figure). Weight loss occurred at day 2. *n*=3. Differences from no weight loss are indicated as c, *P*<0.001.

Pure strain NMRI mice were bred in our own colony and fed economy rodent breeder diet (Special Diet Services, Essex, UK) and water *ad libitum* until they reached a minimum weight of 20 g. Animals were implanted s.c. in the flank with fragments of the MAC16 tumour by means of a trochar, selecting from donor animals with maximal weight loss (Bibby *et al*, 1987). All animal experiments followed a strict protocol, approved by the British Home Office, and the ethical guidelines that were followed met the standards required by the UKCCR guidelines (Workman *et al*, 1998) and involved terminating the animals when the weight loss reached 25–30% of the starting weight or the tumour volume reached 1 cm^3^. Weight loss was evident 10–12 days after tumour transplantation at which time the tumour was just palpable and it took another 3–5 days to reach 25% weight loss. Blood was removed from animals with various extents of weight loss by cardiac puncture under terminal anaesthesia for serum cortisol determination. To establish a time profile for plasma cortisol levels, blood was removed by tail bleed, as indicated in the legend to Figure 1. Animals with established weight loss (5%) were randomised to receive RU38486 (25 mg kg^−1^) daily i.p. in PBS as previously reported (Cheesman and Reilly, 1998), while control animals received PBS alone. Body weight was measured daily and recorded as the change in body weight from the start of the experiment. Tumour volumes were measured daily by means of callipers, and were recorded as a percentage of the starting tumour volume. Non-tumour-bearing mice were administered dexamethasone (66 *μ*g kg^−1^) i.p., while control mice received PBS for 24 h, as reported previously (Jacoby *et al*, 2001). At the end of the experiment, the animals were humanely killed, and the epididymal WAT and BAT were removed for Western blot analysis.

### Materials

Foetal calf serum (FCS) and Dulbecco's modified Eagle's medium (DMEM) were purchased from InVitrogen (Paisley, Scotland). The Cortisol ELISA kit was purchased from Autogen Bioclear UK Ltd (Wiltshire, UK), and assays were performed according to the manufacturer's instructions. A standard curve was produced using murine cortisol. Mouse monoclonal anti-human ZAG antibody with crossreactivity with mouse ZAG was purchased from Santa Cruz (California, USA), while rabbit polyclonal antisera to mouse actin was from Sigma Aldridge (Dorset, UK). Peroxidase-conjugated rabbit anti-mouse antibody was purchased from Dako Ltd (Cambridge, UK). Hybond™ nitrocellulose membranes and enhanced chemiluminescence (ECL) were from American Biosciences (Bucks, UK). Human ZAG was supplied by Bayer Corporation and was purified from Cohn Fraction V effluence derived from large pools of human plasma as described (Russell *et al*, 2004).

### Cell culture

3T3-L1 preadipocytes were grown to confluence in DMEM containing 10% FCS, 1% penicillin/streptomycin, and 1% glutamine in 10% CO_2_ in air at 37°C. At 2 days after the cells reached confluence, differentiation was initiated by addition of growth medium containing 0.5 mM 3-isobutyl-1-methyl-xanthine, 0.25 *μ*M dexamethasone, and 1 *μ*g ml^−1^ insulin, as described (Frost and Lane, 1985), with medium changed every 2 days. This was followed by a further two days in growth medium containing insulin (1 *μ*g ml^−1^), and growth medium was then changed daily. Cells were used experimentally between days 8 and 12, at which time 95% of the cells express the adipocyte phenotype as determined by staining with Oil Red O. For experiments where the media was sampled, the last media change employed DMEM without phenol red. The details of the individual experiments are given in the figure legends. The concentrations of agonists and antagonists employed were based on previous data: isoprenaline (10 *μ*M), ZAG and LMF (0.57 *μ*M), anti-mouse ZAG antibody (1 *μ*g ml^−1^) (Russell *et al*, 2004), and RU38486 (10 *μ*M) (Mulholland *et al*, 2004).

### Western blotting

Samples of adipose tissue and 3T3-L1 adipocytes were homogenised in 0.25 M sucrose, 1 mM HEPES, pH 7.0, and 0.2 M EDTA. Samples of cytosolic protein (10 *μ*g), prepared by centrifugation of the homogenate at 4500 r.p.m. for 10 min, or neat tissue culture medium, were resolved on 12% SDS–PAGE. Proteins were then transferred to nitrocellulose membranes (Hybond™), which had been blocked with 5% Marvel in Tris-buffered saline, pH 7.5, at 4°C overnight. Both primary and secondary antibodies were used at a dilution of 1 in 1000. Incubation with the primary antibody was carried out overnight at 4°C, and, following four 15-min washes with 0.1% Tween in PBS, incubation with the secondary antibody was performed for 1 h at room temperature. Development was by ECL. Blots were subjected to densitometric analysis using ‘Phoretix ID Advanced’ software. Equal loading was confirmed by Ponceau S staining of the membranes and actin blotting, which was performed on the same samples at the same time as ZAG.

### Statistical analysis

Results are expressed as mean±s.e.m. Differences were determined by one-way ANOVA, followed by Tukey–Kramer multiple comparison test. *P*-values less than 0.05 were considered to be significant.

## RESULTS

**Figure 2:**
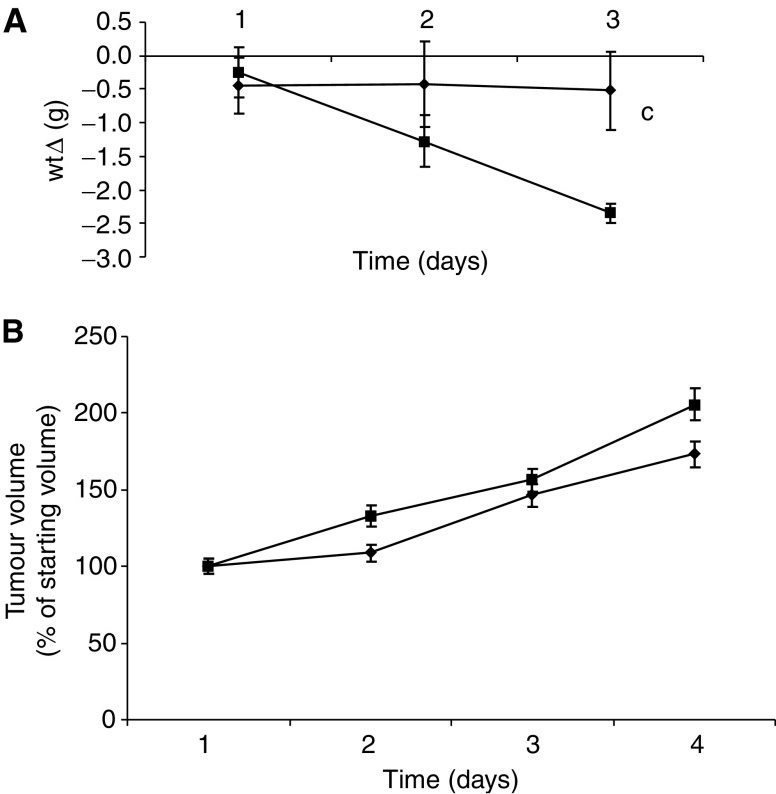
Effect of RU38486 (25 mg kg^−1^ i.p. daily) (♦) on changes in body weight (**A**) and tumour volume (**B**) in mice bearing the MAC16 tumour. Differences from PBS control (▪) are indicated as c, *P*<0.001. Number of animals in each group, *n*=6. Day 1 is 11 days after transplantation when the mice start to lose weight.

**Figure 3:**
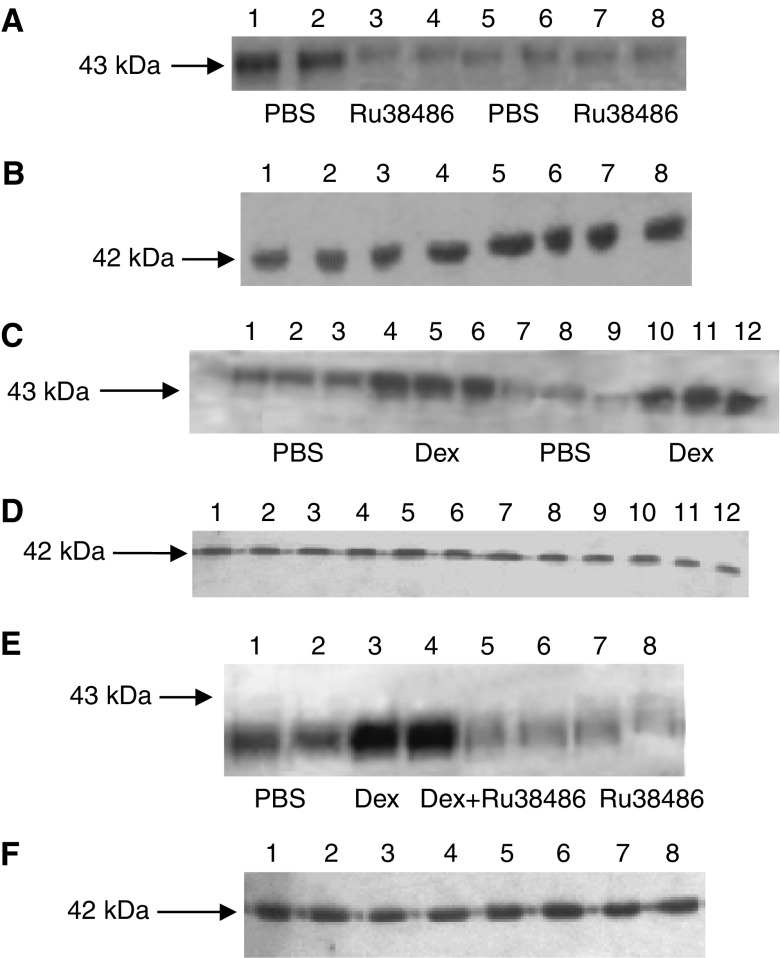
(**A**) Western blot of ZAG expression in WAT of mice bearing the MAC16 tumour treated with PBS (lanes 1–2) or RU38486 (25 mg kg^−1^) (lanes 3–4) and in non-tumour-bearing mice treated with either PBS (lanes 5–6) or RU38486 (25 mg kg^−1^ i.p. daily for 4 days) (lanes 7–8). The experiment was initiated 11 days after tumour transplantation when the average weight loss was 5%. (**B**) Actin loading control for the blot shown in (**A**). (**C**) Western blot of ZAG expression in epididymal WAT (lanes 1–6) and BAT (lanes 7–12) of non-tumour-bearing NMRI mice 24 h after administration of PBS (lanes 1–3 and 7–9) or dexamethasone (66 *μ*g kg^−1^ i.p. daily) (lanes 4–6 and 10–12). *n*=6. (**D**) Actin loading control for the blot shown in (**A**). (**E**) Western blot of ZAG expression in WAT of NMRI mice treated with PBS (lanes 1–2), dexamethasone (lanes 3–4), dexamethasone+RU38486 (25 mg kg^−1^) (lanes 5–6) or RU38486 (25 mg kg^−1^) (lanes 7–8). *n*=6. (**F**) Actin loading control for the blot shown in (**E**).

The effect of weight loss in mice bearing the MAC16 tumour on serum cortisol levels over the course of the development of cachexia is shown in Figure 1. Serum cortisol concentrations increased in parallel with the weight loss (Figure 1A) and were elevated within 1 day of weight loss (Figure 1B), suggesting that it could be responsible for the increased ZAG expression seen in adipose tissue of cachectic mice (Bing *et al*, 2004). To investigate this possibility, mice bearing the MAC16 tumour and with an average weight loss of 5% were treated with the glucocorticoid receptor antagonist RU38486 (25 mg kg^−1^) and the effect on loss of body weight and tumour volume was determined (Figure 2). Treatment with RU38486 completely attenuated loss of body weight in mice bearing the MAC16 tumour (Figure 2A), while having no effect on tumour volume (Figure 2B). Western blot analysis showed that ZAG expression in WAT was upregulated in mice bearing the MAC16 tumour (Figure 3) and was downregulated by 50% (*P*<0.001) in animals treated with RU38486. To confirm that circulating glucocorticoids could be responsible for the increased ZAG expression in mice bearing the MAC16 tumour, normal mice were administered dexamethasone (66 *μ*g kg^−1^) by i.p. injection, twice at 12-h intervals, and killed at 24 h. Western blotting showed about a two-fold increase in ZAG expression in both WAT and BAT (*P*<0.001) (Figure 3C and E), and the effect was completely attenuated by the glucocorticoid receptor antagonist RU38486 (*P*<0.001 from dexamethasone alone) (Figure 3E), confirming that it was mediated through the glucocorticoid receptor.

**Figure 4:**
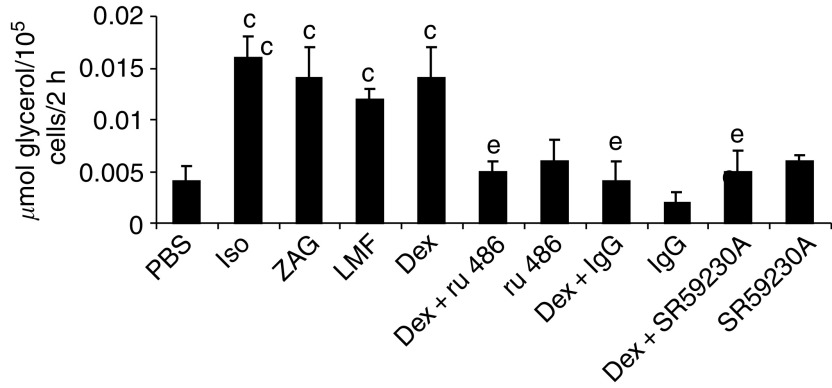
Effect of various treatments on lipolysis in 3T3-L1 adipocytes, determined by the release of glycerol over a 24-h period. 3T3-L1 cells were treated with isoprenaline (10 *μ*M), ZAG and LMF (0.57 *μ*M), dexamethasone alone (1.68 *μ*M) or combined with RU38486 (10 *μ*M), anti-mouse ZAG antibody (1 *μ*g ml^−1^), EPA (50 *μ*M) or SR59230A (10 *μ*M) and the various inhibitors alone in a total volume of 2 ml. The inhibitors were added 2 h prior to the agonists. Glycerol release was determined by an enzymatic/spectrascopic method as described (Wieland, 1974). The values shown are for *n*=6. Differences from control are indicated as c, *P*<0.001, while differences from dexamethasone alone are indicated as e, *P*<0.01.

To investigate the mechanism of the regulation of ZAG expression by glucocorticoids, further experiments were carried out *in vitro* using 3T3-L1 adipocytes. The effect of various treatments on glycerol release over a 24-h period is shown in Figure 4. Dexamethasone (1.68 *μ*M) stimulated lipolysis to the same extent as ZAG and LMF (0.57 *μ*M), and these values were similar to those of the positive control (isoprenaline; 10 *μ*M). The lipolytic activity of dexamethasone was attenuated by RU38486 (10 *μ*M), confirming that it was working through the glucocorticoid receptor. In addition, lipolytic activity was blocked by anti-ZAG monoclonal antibody (1 *μ*g ml^−1^), suggesting that the action of dexamethasone on lipolysis was mediated through the induction of ZAG expression. The selective *β*3-AR receptor antagonist (Nisoli *et al*, 1996) SR59230A (10 *μ*M) also completely attenuated the action of dexamethasone, suggesting that the ZAG produced moved into the medium and acted extracellularly though *β*3-AR.

**Figure 5:**
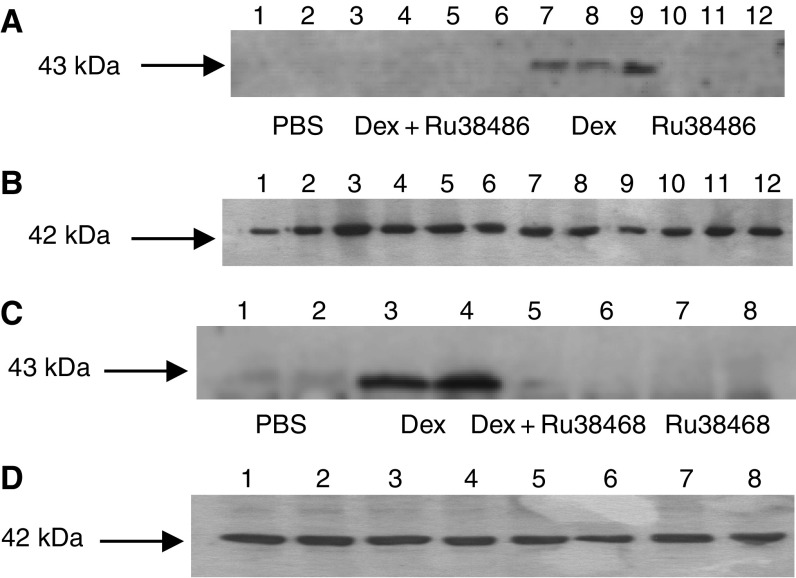
(**A**) Western blot of ZAG expression in 3T3-L1 adipocytes from cells treated with PBS (lanes 1–3), dexamethasone (1.68 *μ*M)+RU38486 (lanes 4–6), dexamethasone (1.68 *μ*M) (lanes 7–9) or RU38486 (10 *μ*M) (lanes 10–12) for 24 h. RU38486 was added 2 h prior to dexamethasone. Adipocytes were washed three times in PBS to remove traces of media prior to homogenisation. *n*=6. (**B**) Actin loading control for the blot shown in (**A**). (**C**) Western blot of ZAG expression in media from 3T3-L1 adipocytes treated with PBS (lanes 1 and 2), dexamethasone (1.68 *μ*M) (lanes 3 and 4), dexamethasone (1.68 *μ*M)+RU38486 (10 *μ*M) (lanes 5 and 6) or RU38486 (10 *μ*M) (lanes 7 and 8) for 24 h. RU38486 was added 2 h prior to dexamethasone. *n*=6. (**C**, **D**) Actin loading control for the blot shown in (**C**).

**Figure 6:**
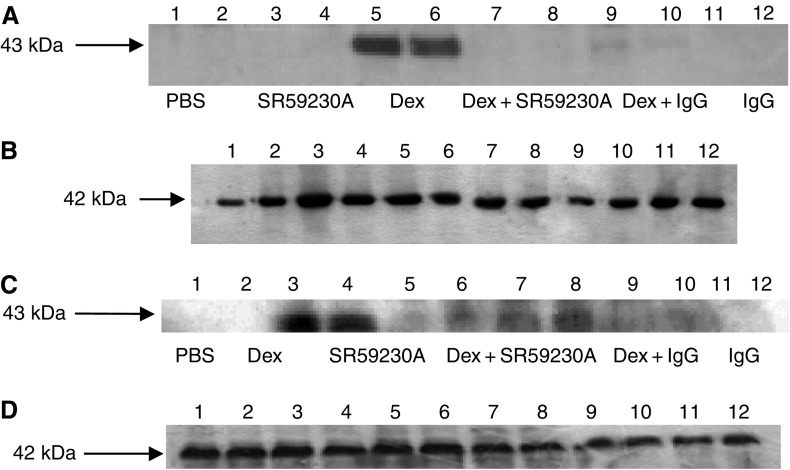
Expression of ZAG in cells (**A**) and medium (**C**) of 3T3-L1 adipocytes treated with PBS (lanes 1 and 2), SR59230A (10 *μ*M) (lanes 3 and 4) (lanes 5 and 6 in (**C**)), dexamethasone (1.68 *μ*M) (lanes 5 and 6) (lanes 3 and 4 in (**C**)), dexamethasone (1.68 *μ*M)+SR59230A (10 *μ*M) (lanes 7 and 8), dexamethasone (1.68 *μ*M)+anti-ZAG antibody (1 *μ*g ml^−1^) (lanes 9 and 10) and anti-ZAG antibody (1 *μ*g ml^−1^) (lanes 11 and 12) for 24 h as determined by Western blotting. The inhibitors were added 2 h prior to the agonists. The actin loading controls are shown in (**B**) and (**D**).

**Figure 7:**
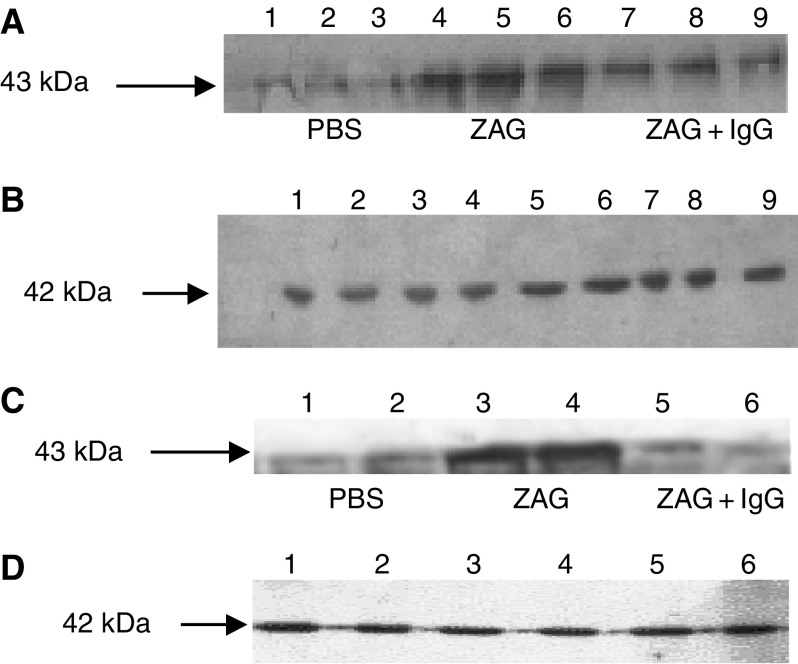
(**A**) Effect of ZAG on expression of ZAG in 3T3-L1 adipocytes and in medium (**C**) treated with PBS (lanes 1–3 in (**A**) and lanes 1 and 2 in (**C**)), ZAG (0.57 *μ*M) (lanes 4–6 in (**A**) and lanes 3 and 4 in (**C**)) and ZAG (0.57 *μ*M)+anti-ZAG antibody (1 *μ*g ml^−1^) (lanes 7–9 in (**A**) and lanes 5 and 6 in (**C**)) for 24 h. The antibody was added 2 h prior to ZAG. The adipocytes were washed three times with PBS prior to homogenisation to remove traces of extracellular ZAG. *n*=6. The actin loading controls are shown in (**B**) and (**D**).

**Figure 8:**
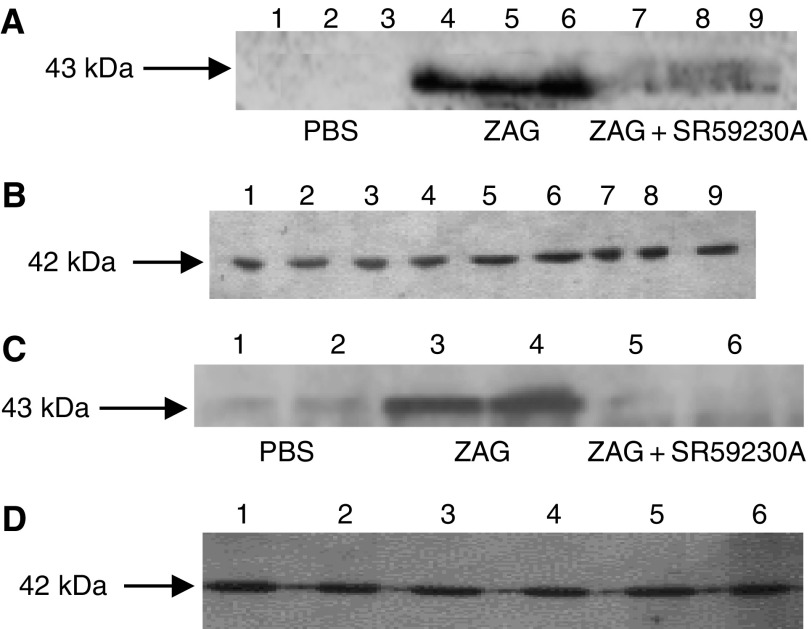
(**A**) Effect of ZAG on expression of ZAG in 3T3-L1 adipocytes and in medium (**C**) treated with PBS (lanes 1–3 in (**A**) and 1 and 2 in (**C**)), ZAG (0.57 *μ*M) (lanes 4–6 in (**A**) and 3 and 4 in (**C**)) and ZAG (0.57 *μ*M)+SR59230A (10 *μ*M) (lanes 7–9 in (**A**) and 5 and 6 in (**C**)) for 24 h. SR59230A was added 2 h prior to ZAG. The adipocytes were washed three times in PBS prior to homogenisation to remove traces of extracellular ZAG. The actin loading controls are shown in (**B**) and (**D**).

To confirm this hypothesis, ZAG expression in 3T3-L1 adipocytes and media in response to dexamethasone and various antagonists was determined by Western blotting. As expected from *in vivo* data (Figure 3), dexamethasone stimulated a three-fold increase in ZAG expression in both cells and medium (Figure 5) (*P*<0.001 from PBS), and this was attenuated by RU38486 (*P*<0.001 from dexamethasone). Interestingly, ZAG expression was also completely downregulated (*P*<0.001) when dexamethasone was combined with SR59230A, suggesting a role for a *β*3-AR in the induction of ZAG expression (Figure 6). Anti-ZAG antiserum also attenuated dexamethasone-induced ZAG expression (*P*<0.001), suggesting that ZAG itself may be involved in the increased gene transcription. This conclusion was substantiated from studies with ZAG (Figure 7). Zinc-*α*_2_-glycoprotein induced increased expression of ZAG in both 3T3-L1 adipocytes and in medium, and this was attenuated by pretreatment (2 h prior to ZAG) with anti-ZAG antibody (Figure 7A and C). Zinc-*α*_2_-glycoprotein expression was also attenuated by SR59230A (Figure 8) (*P*<0.001), suggesting that induction of ZAG is mediated through a *β*3-AR.

## DISCUSSION

Glucocorticoids have been investigated as potential mediators of the process of cancer cachexia, since serum levels are elevated in cachectic cancer patients compared with those without cachexia (Knapp *et al*, 1991), and they have the potential for stimulation of muscle proteolysis (Wing and Goldberg, 1993) and adipose tissue lipolysis (Lacasa *et al*, 1988). However, this possibility has largely been discounted, since wasting of body tissues was not prevented by adrenalectomy in mice bearing the MG101 sarcoma (Svaninger *et al*, 1987), while the glucocorticoid receptor antagonist RU38486 had no effect on the development of cachexia in rats bearing the Yoshida AH-130 hepatoma (Llovera *et al*, 1996). It is not clear why cachexia in these two models should be independent of glucocorticoids, since in mice bearing the MAC16 tumour serum levels of cortisol increased with increasing weight loss, and the development of cachexia was attenuated by the glucocorticoid receptor antagonist RU38486, with a concomitant reduction in ZAG expression in adipose tissue. This suggests that elevation of glucocorticoid levels during the development of cachexia is responsible for the increased ZAG expression in adipose tissue, which in turn leads to an increased lipid mobilisation and utilisation (Russell and Tisdale, 2002). That glucocorticoids are able to enhance ZAG expression *in vivo* has been confirmed by the systemic administration of dexamethasone, suggesting that the increased ZAG expression seen in adipose tissue of mice bearing the MAC16 tumour (Bing *et al*, 2004) is probably due to circulating cortisol. The only other potential mediator of the increased ZAG expression in cachexia is interferon-*γ* (IFN*γ*), which has been shown to strongly upregulate expression in human epithelial cell lines (Brysk *et al*, 1997), and has been suggested to be responsible for the development of cachexia in the Lewis lung tumour model (Matthys *et al*, 1991b). However, cachexia in mice bearing the MAC16 tumour is not thought to be cytokine mediated (Mulligan *et al*, 1992), and is not associated with the development of anorexia seen in mice inoculated with CHO cells transfected with the IFN*γ* gene (Matthys *et al*, 1991a).

Dexamethasone has been shown to decrease the body weight of rats, decrease the amount of adipose tissue and increase the catecholamine-stimulated lipolysis (Pedersen *et al*, 2003). Exposure of adipose tissue fragments to dexamethasone has been shown to lead to enhanced lipolytic and cyclic AMP responses to isoprotorenol (Lacasa *et al*, 1988). The permissive effect of the steroid was prevented by the glucocorticoid receptor antagonist RU38486. The current study shows that dexamethasone directly stimulated lipolysis in 3T3-L1 adipocytes, and that was associated with an increased ZAG expression in cells and medium. The lipolytic effect of ZAG was attenuated by RU38486, as was ZAG expression. This suggests that the permissive effect of glucocorticoids towards lipolysis may result from an increase in ZAG expression. Dexamethasone has been shown to stimulate adenylate cyclase catalytic activity and response to GTP in fat cell membranes (Lacasa *et al*, 1988). Zinc-*α*_2_-glycoprotein has also been shown to cause a three-fold increase in lipolytic response to isoprenaline, arising from changes in GTP-binding proteins (G-proteins), with an increased expression of G*α*s and a decreased expression of G*α*i in adipose tissue (Islam-Ali *et al*, 2001).

The mechanism by which glucocorticoids stimulate ZAG expression appears complex, since expression was attenuated by both a *β*3-AR antagonist and anti-ZAG antibody, as well as the glucocorticoid receptor antagonist RU38486. This suggests that dexamethasone induces ZAG initially through the glucocorticoid receptor, and the ZAG moves into the medium and stimulates further ZAG production via a *β*3-AR. Certainly the *β*3-AR agonist, BRL37344 has been shown to increase ZAG mRNA levels in 3T3-L1 adipocytes (Bing *et al*, 2004). Glucocorticoids have a complex effect on *β*3-AR expression in brown adipocytes (Bakopanos and Silva, 2002). They rapidly and directly inhibit transcription, but they also induce a rapidly turned-over protein that stimulates gene transcription. It is therefore possible that glucocorticoids induce *β*3-AR expression in 3T3-L1 adipocytes, amplifying the induction of ZAG expression. Zinc-*α*_2_-glycoprotein itself has been shown to induce ZAG expression in 3T3-L1 adipocytes, and this effect is attenuated by anti-ZAG antibody and SR59230A, confirming that it is mediated through a *β*3-AR, as for dexamethasone. Even though human ZAG was used in these experiments it was capable of inducing murine ZAG, suggesting that the mechanism of regulation in the two species is probably identical. The mechanism by which stimulation of *β*3-AR leads to an increased ZAG expression is not known. The ability of ZAG to induce its own expression in adipocytes would explain why the effect on lipolysis *in vivo* is much greater than expected from the *in vitro* lipolytic effect (Russell *et al*, 2004). It would also explain why relatively small changes in plasma ZAG may have profound effects on lipid mobilisation. These results suggest that induction of ZAG expression may play an important role in the action of glucocorticoids.
